# Long-Term Efficacy of Low-Intensity Single Donor Fecal Microbiota Transplantation in Ulcerative Colitis and Outcome-Specific Gut Bacteria

**DOI:** 10.3389/fmicb.2021.742255

**Published:** 2021-11-17

**Authors:** Rongrong Ren, Xuefeng Gao, Yichao Shi, Jianfeng Li, Lihua Peng, Gang Sun, Zikai Wang, Bin Yan, Junli Zhi, Yunsheng Yang

**Affiliations:** ^1^Department of Gastroenterology and Hepatology, The First Medical Center, Chinese PLA General Hospital, Beijing, China; ^2^Department of Gastroenterology and Hepatology, Shenzhen University General Hospital, Shenzhen, China; ^3^Central Laboratory, Shenzhen Key Laboratory of Precision Medicine for Hematological Malignancies, Shenzhen University General Hospital, Shenzhen, China

**Keywords:** ulcerative colitis, low-intensity FMT, long-term efficacy, gut bacteria, 16s rRNA

## Abstract

**Aims**: To assess the long-term efficacy and safety of single-donor, low-intensity fecal microbiota transplantation (FMT) in treating ulcerative colitis (UC), and to identify the outcome-specific gut bacteria.

**Design:** Thirty-one patients with active UC (Mayo scores ≥ 3) were recruited, and all received FMT twice, at the start of the study and 2∼3 months later, respectively, with a single donor and a long-term follow-up. The fecal microbiome profile was accessed via 16S rRNA sequencing before and after FMT.

**Results:** After the first FMT, 22.58% (7/31) of patients achieved clinical remission and endoscopy remission, with the clinical response rate of 67.74% (21/31), which increased to 55% (11/20) and 80% (16/20), respectively, after the second FMT. No serious adverse events occurred in all patients. During 4 years of follow-up, the mean remission period of patients was 26.5 ± 19.98 m; the relapse rate in the 12 remission patients was 33.33% within 1 year, and 58.3% within 4 years. At baseline, UC patients showed an enrichment in some proinflammatory microorganisms compared to the donor, such as *Bacteroides fragilis*, *Clostridium difficile*, and *Ruminococcus gnavus*, and showed reduced amounts of short-chain fatty acid (SCFA) producing bacteria especially *Faecalibacterium prausnitzii.* FMT induced taxonomic compositional changes in the recipient gut microbiota, resulting in a donor-like state. Given this specific donor, UC recipients with different outcomes showed distinct gut microbial features before and after FMT. In prior to FMT, relapse was characterized by higher abundances of *Bacteroides fragilis* and *Lachnospiraceae incertae sedis*, together with lower abundances of *Bacteroides massiliensis*, *Roseburia*, and *Ruminococcus*; *Prevotella copri* was more abundant in the non-responders (NR); and the patients with sustained remission (SR) had a higher abundance of *Bifidobacterium breve*. After FMT, the NR patients had a lower level of *Bifidobacterium* compared to those with relapse (Rel) and SR, while a higher level of *Bacteroides* spp. was observed in the Rel group.

**Conclusion:** Low-intensity single donor FMT could induce long remission in active UC. The gut microbiota composition in UC patients at baseline may be predictive of therapeutic response to FMT.

## Introduction

Ulcerative colitis (UC) is a chronic idiopathic inflammatory bowel disorder, which is difficult to cure and easy to relapse. It has become a global disease with a high prevalence in western countries, and the growing incidence in newly industrialized countries (Ng et al., 2018). The exact pathogenesis of UC is thought to be a multi-factor disease, which is a result of the interaction between host susceptibility genes, environment, diet, immunity, intestinal barrier, and gut microbiota (Ramos and Papadakis, 2019). A large body of evidence has revealed the intestinal microbiome playing a critical role in the pathogenesis of UC, and has shown major shifts of intestinal flora in UC patients, such as reduced bacterial diversity, higher abundance of Proteobacteria and lower abundance of Firmicutes (Sokol and Seksik, 2010; Lane et al., 2017; Franzosa et al., 2019). However, the perturbation of specific microbes involved in UC pathogenesis have not been identified.

Several clinical trials have been performed to treat UC by manipulating the intestinal microbiota through probiotics, prebiotics, synbiotics, and antibiotics (Ghouri et al., 2014; Asto et al., 2019; Xi et al., 2021). However, evidence supporting significant therapeutic effects for UC remains limited. Fecal microbiota transplantation (FMT) is a powerful way to manipulate gut microbiota, which has been proven to be an effective treatment for *Clostridium difficile* infection (CDI). So far, only five randomized controlled trials (RCTs) studies were published regarding FMT treatment for UC (Supplementary Table 1), including four for adult patients and one for pediatric patients, and three of which reported higher remission rates [24%(Moayyedi et al., 2015), 27%(Paramsothy et al., 2017), and 32%(Costello et al., 2019)] in patients received high-intensity FMT compared to placebo (5, 8, and 9%, respectively). The pediatric RCT study also showed a higher composite clinical outcome of FMT group versus placebo group (91.7% vs. 50% at 6 weeks) (Pai and Popov, 2017; Pai et al., 2021). One RCT study reported a negative result in treating UC, with remission rates 30.4% vs. 20.0% compared to the control (*p* = 0.51) (Rossen et al., 2015). The uniform protocols of FMT administration have not been achieved, and there is a paucity of data on the long-term durability and safety of FMT in patients with active UC especially those with moderate to severe disease. In addition, most previous studies including RCTs performed FMT with high intensities, which increases the practical barriers. In this study, we assessed the long-term efficacy and safety of single-donor, low-frequency FMT in the treatment of UC, and analyzed the intestinal microbiota characteristics associated with different therapeutic outcomes.

## Materials and Methods

### Trial Design

This was a single-center, historical control trial of FMT for the treatment of active UC. Patients received the initial (F0) and second (F1) treatments with time interval of 2 months. The follow-up clinical and endoscopy examinations were performed at F1 and 4 months after F0, which were marked as F2.

### Enrollment

Eligible patients fulfilled the following criteria: established UC according to clinical symptoms, colonoscopy, and pathology; Mayo scores ≥ 3; Mayo endoscopic scores ≥ 2; initial onset cases without any treatment, or, subjects refractory or intolerant to the existing treatments including 5-ASA, glucocorticoids, immunosuppressants and biologics; a 1-week washout period before treatment if the patients were exposed to antibiotics, probiotics or other medicines that could influence intestinal flora; and the ability to provide informed consent. Patients were excluded if they underwent long-term prokinetic treatment to control diarrheal symptom, had a history of colectomy or other intestinal surgery, had a concomitant *C. difficile* infection or infection with another enteric pathogen, had severe congenital or acquired immunodeficiency disease, had a progressive severe disease except for UC that required hospitalization, or were pregnant or lactating.

### The Cohort

The enrolled patients completed general questionnaires for demographic information including age, gender, weight, body mass index, and Inflammatory Bowel Disease Questionnaire score (IBDQ, a validated disease-specific quality of life measure; scores range from 0 to 224 with a higher score indicating better quality of life) (Guyatt et al., 1989). Clinical syndromes were recorded, including body temperature, daily defecation frequency, hematochezia, stool consistency, abdominal pain, abdominal distension, etc. Lab test included hemoglobin (Hb), number of leukocytes, erythrocyte sedimentation rate (ESR), c-reactive protein (CRP), platelet count, etc., were examined. Endoscopic performance, baseline Mayo scores, clinical Mayo scores and endoscopic Mayo scores were documented.

### Donor

We screened self-perceived healthy volunteers heavily to acquire eligible donors via a preliminary screening questionnaire, laboratory examinations. These donor candidates reported their medical history and lifestyle habits via questionnaires to exclude any exposure to infectious agents or risky behaviors (e.g., sexual preference). They underwent serology screening tests for HIV, hepatitis A, B, C, and E, syphilis, Epstein–Barr virus, cytomegalovirus, rotavirus. Stool culturing was performed for enteric pathogens including *Escherichia coli O157*, *Salmonella* spp., *Shigella* spp., *Campylobacter* spp., *Staphylococcus aureus*, *Yersinia*, *Vibrio parahaemolyticus*, *Vibrio cholerae*, *Candida albicans*, *Clostridium difficile* toxin A/B, as well as ova and parasites. In addition, physical examination, electrocardiogram, chest X-ray, urea breath test, as well as blood tests were also performed to exclude gastrointestinal, or non-gastrointestinal disorders. All eligible donors had negative results for these tests and examinations. Among the donor candidates we selected, one 12-years old boy showed outstanding therapeutic outcomes in treating UC patients in our previous report (Ren et al., 2015). In order to further define beneficial donor-specific and microbial content-specific effects, we only used the material from this “super-donor” candidate for FMT in this study. The donor has signed informed consent for each donation.

### Fecal Microbiota Transplantation Interventions and Follow-Up

The donor did not use antibiotics, probiotics or other agents that could influence intestinal flora and had no travel history during the feces donation time, and he was requested to repeat screening every 3 months. Donor feces were collected on the day of treatment into a sterile medical container in a special bathroom and stored on ice, then sent to the laboratory within 1 h. Then, a total of 350 to 400 ml filtered stool suspension was obtained from a mixture of 100 g specimen stool and 500 ml sterile saline solution and immediately transferred to the endoscopy center on ice for later use. FMT was administrated via colonoscopy or colonoscopy combined with gastroscopy. Patients were pretreated with 2L bowel lavage solution (polyethylene glycol electrolyte disperses) on the morning of the treatment. Patients underwent a routine colonoscopy examination, during which biopsy specimens were obtained. An endoscopic spray tube (model:AF-2416PB, Olympus, Japan) was inserted into the ileum through the biopsy channel of the colonoscope, and then approximately 300 ml of stool suspension was infused with a syringe into the tube as the colonoscope was slowly retracted. If a combined gastroscopic approach was applied, the tube was placed in the duodenal descending portion to deliver the stool suspension (approximately 80∼100 ml). After delivery, patients stayed in bed for at least 45 to 60 min.

### Measures and Outcomes

The clinical syndromes, lab indexes, IBDQ, Mayo scores (MS) and endoscopic Mayo scores (EMS) were recorded at F0, F1, and F2. Adverse effects were recorded after each FMT and during the follow-up period.

The primary end points of the study included clinical remission [defined as a total Mayo score ≤ 2, with no individual sub-score >1 (Rutgeerts et al., 2005; D’Haens et al., 2007; Carbonnel et al., 2016)], clinical response [defined as a decrease in the Mayo score of at least 3 points and at least 30%, with an accompanying decrease in the sub-score for rectal bleeding of at least 1 point or an absolute rectal-bleeding sub-score of 0 or 1 (Rutgeerts et al., 2005; D’Haens et al., 2007; Carbonnel et al., 2016)], and endoscopy remission [defined as an absolute sub-score for endoscopy of 0 or 1 (Rutgeerts et al., 2005; D’Haens et al., 2007; Paul et al., 2013)] at the end of the follow-up period. Patients were followed for up to 2∼5 years after inclusion and long-term clinical remission (or sustained remission) was defined as clinical remission without the need of rescue therapy at or more than 2 years. All the remission and response here were steroid-free.

The secondary end points included adverse events, changes in IBDQ score, CRP, ESR, Hb, weight, BMI, as well as relapse rate during long-term follow-up. The relapse rate was defined as the proportion of relapsed patients in patients with remission during long-term follow-up.

### Specimen Collection and Microbiota Profiling

Fecal samples were collected from patients at three time points and from the corresponding donor on the day of treatment. Samples were divided into sterile tubes, stored in a −20°C freezer immediately after production and subsequently transferred to −80°C within 24 h. DNA was isolated using QIAamp^®^ DNA Stool Mini Kit (Qiagen, Valencia, CA, United States) from fecal samples, and then its concentration was measured by Nanodrop2000 instrument (Thermo Scientific, United States) and molecular size was estimated by agarose gel electrophoresis. 16S rRNA genes of V3-V4 regions were amplified and raw sequencing data were processed with the Illumina MiSeq platform as described previously (Ren et al., 2018).

USEARCH pipeline was applied to denoise the raw sequences, remove chimeras, and produce operational taxonomic units (OTUs) (Edgar, 2010). Sequences were assembled using –*fastq_mergepairs* command with default parameters, and quality trimmed using –*fastq_filter* command with a –*fastq_maxee* set at 1.0. The assembled sequences were clustered into zero-radius OTUs by using unoise3 (Edgar, 2013) algorithm with the minimum abundance cut-off (–*minsize*) set at 8. Taxonomic assignments to the OTUs were performed using SINTAX with Ribosomal Database Project (RDP) 16S training set as the reference database (Cole et al., 2014).

### Statistical Analysis

The clinical data were analyzed by GraphPad Prism v7.0 (GraphPad Software Inc., CA, United States) or SPSS v23.0 software (SPSS, Chicago, IL, United States). Categorical data were analyzed using Pearson’s χ^2^ or Fisher’s exact test. Continuous data were analyzed using *t* tests with significance defined as *p* < 0.05.

Statistical analysis of the microbiota profiles was performed by using Calypso (v 8.6.4) (Zakrzewski et al., 2017). Taxa that have less than 3,000 read counts or 2% relative abundance across all samples were excluded from analysis. In order to account for the non-normal distribution of taxonomic counts data, the sequences of OTUs were normalized via Cumulative-sum scaling (CSS) followed by log2 transformation. Alpha diversity was quantified at the OTU level using the Shannon’ and Chao1 indexes indices, testing for significant differences with analysis of variance (ANOVA) followed by Tukey *post hoc* test. For beta diversity, we performed principal coordinates analysis (PCoA) using Bray-Curtis dissimilarity at the OTU level, and determined significant differences among groups using permutational multivariate analysis of variance (PERMANOVA). Three or more group-wise comparisons were performed by Kruskal–Wallis testing on the relative abundance of fecal bacteria. Further pairwise Mann-Whitney test was carried out between all groups to assess relevant signatures.

## Results

### Patients

**FIGURE 1:**
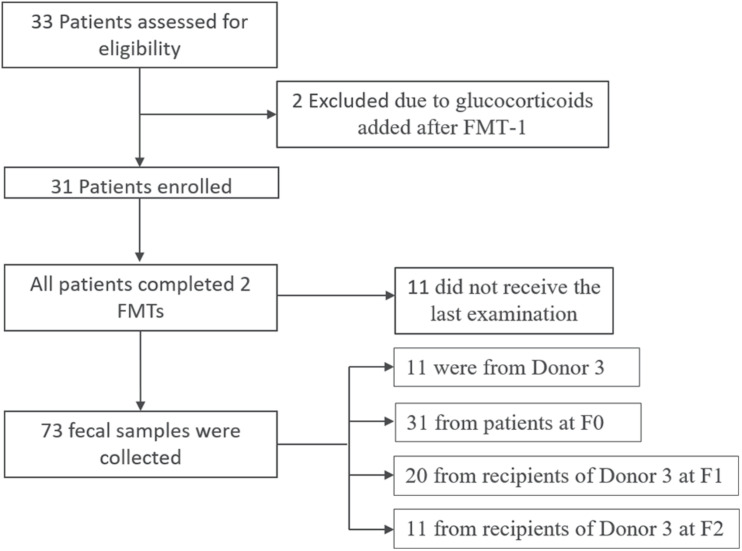
Flow of patients in the trial.

An overview of the recruitment process is shown in Figure 1. From November 2014 to May 2017, 33 patients diagnosed with active UC were recruited. Two patients were excluded in the subsequent analysis due to glucocorticoids administration after the first FMT. Eleven patients did not receive the last examination after the second FMT. The baseline characteristics of the enrolled patients are presented in Table 1. The 31 enrolled patients included 19 males and 12 females, with an average age of 36 ± 12.39 years (range 14 to 62 years) and an average disease course of 4.44 ± 4.52 years (range 2 months to 20 years). Among all of the UC patients according to the Montreal classification (Saidani et al., 2019), 77.4% (24 cases) had extensive UC (E3, pancolitis), 16.1% (5 cases) had left sided UC (E2, distal UC), and 6.5% (2 cases) had ulcerative proctitis (E1) (Satsangi et al., 2006). Three treatment-naïve patients with new-onset UC, and the rest had a medication history including the use of mesalamine, glucocorticoids, immunosuppressants (azathioprine) or tumor necrosis factor antagonists (infliximab). Each patient received FMT treatment twice with an interval of 2–3 months. The medications of the UC patients before and after FMT are presented in Supplementary Tables 2, 3.

**TABLE 1:** Baseline characteristics of enrolled UC patients.

**Characteristic**	**Patients (*n* = 31)**
Gender, n (male/female)	19/12
Age, mean ± SD (range)	36 ± 12.39 (14∼62)
Disease course (year)	4.44 ± 4.52
Height, mean ± SD (m)	1.70 ± 0.11
Weight, mean ± SD (kg)	56.69 ± 12.73
BMI, mean ± SD	19.76 ± 3.59
Montreal classification *, n (E1/E2/E3)	2/5/24
Mayo scores, mean ± SD	9.58 ± 2.63
Severity, n (mild/moderate/severe)	3/12/16

*E3, extensive UC (pancolitis); E2, left sided UC (distal UC); E1, ulcerative proctitis; according to the Montreal classification.

### Responses

**FIGURE 2:**
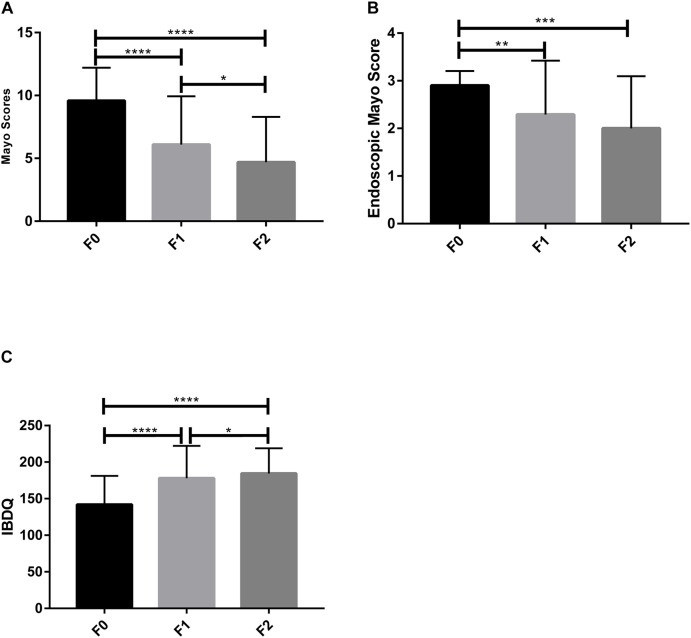
Clinical indexes of patients. **(A)** Mayo score, **(B)** Endoscopic Mayo score, and **(C)** IBDQ score were measured to evaluate the FMT therapeutic outcomes. F0, before FMT; F1, 2∼3 months after the first FMT, or before the second FMT; F2, 2∼3 months after the second FMT. *p < 0.05, **p < 0.01, ***p < 0.001, ****p < 0.0001 (Paired *t*-test with GraphPad Prism 7.00).

Overall, the Mayo scores and endoscopic Mayo scores significantly decreased in UC patients compared to the baseline (*p* < 0.05) (Figures 2A,B). After the first FMT (FMT-1), the clinical remission rate and endoscopic remission rate were 22.58% (7/31), and the clinical response rate was 67.74% (21/31). After the second FMT (FMT-2), only 20 patients received the last examination after the second FMT, and the clinical remission rate and endoscopic remission rate rose to 60% (12/20), and the clinical response rate increased to 80% (16/20).

The efficacy of FMT was diverse in patients with different symptom severity showed. After two FMT treatments, the clinical remission rate and response rate of moderate UC were much higher than mild and severe UC. It is worth mentioning that all the moderate UC patients were responded to FMT treatment (Table 2). Of all the patients, 11 were administrated in the route of colonoscopy combined with gastroscopy, and 20 were delivered only through colonoscopy. After two FMT treatments, the clinical remission rates and clinical response rates were 27.3% (3/11) vs. 45% (9/20), and 63.6% (7/11) vs. 70% (14/20) (Table 2). The intestinal mucosa lesions and histopathological images of patients improved to different extents after two FMT treatments (Supplementary Figure 1). The clinical responses of E3 (pancolitis), E2 (distal UC), and E1 (ulcerative proctitis) patients were 83.3% (20/24), 80% (4/5), and 50% (1/2), respectively (Table 2). There was an interesting phenomenon that the lesions in the rectum and/or sigmoid colon persisted in some E3 patients.

**TABLE 2:** Clinical efficacy after two FMT treatments under different conditions.

**Clinical conditions**		**Clinical remission rates**	**Clinical response rates**
severity	Mild UC	33.3%(1/3)	33.3%(1/3)
	Moderate UC	58.3%(7/12)	100%(12/12)
	Severe UC	25%(4/16)	75%(12/16)
Administration routes	Colonoscopy combined with gastroscopy (*n* = 11)	27.3%(3/11)	63.6%(7/11)
	Colonoscopy only (*n* = 20)	45%(9/20)	70%(14/20)
Montreal classification	E3 (*n* = 24)	41.7%(10/24)	83.3%(20/24)
	E2 (*n* = 5)	40%(2/5)	80%(4/5)
	E1 (*n* = 2)	0	50%(1/2)

*In this table, the 11 patients who did not receive the examination at F2, were analyzed based on the assessments at F1.*

Fecal microbiota transplantation treatment significantly decreased the defecation frequency, improved hematochezia, and increased the body weight and BMI of the UC patients after FMT-1 (Table 3). ESR, CRP and platelets decreased significantly after FMT-1 and FMT-2 (*p* < 0.05); there was no significant difference in Hb before and after FMT, though it had an increasing trend (Table 3). The total IBDQ score increased significantly after FMT (*p* < 0.0001) (Figure 2C).

**TABLE 3:** Clinical variables before and after FMT.

**Clinical variables**	**F0 (*n* = 31)**	**F1 (*n* = 31)**	**F2 (*n* = 17)**	***p* value**
Hematochezia scores	2.191.11	1 ± 1.13****	0.53 ± 1.007****	<0.0001
Stool frequency	8.236.17	4.29 ± 4.31**	2.82 ± 2.68**	0.0005
ESR (mm/h)	22.5619.69	15.48 ± 17.2	14.88 ± 15.55	0.256
CRP (mg/dl)	1.832.09	1.23 ± 1.84	0.67 ± 0.71*	0.116
High ESR, % (n/30)	46.43% (13/28)	28.57% (8/28)	23.53% (4/17)	0.211
High CRP, % (n/30)	51.61% (16/31)	29.03% (9/31)*	23.53% (4/17)*	0.081
Platelet	335.6136.2	316.5 ± 122.9	276.9 ± 93.82*	0.312
Hb	111.918.4	116.7 ± 24.78	116.4 ± 24.68	0.664
Weight (kg)	56.6912.73	58.86 ± 13.19**	58.32 ± 13.97**	0.802
BMI	19.763.59	20.49 ± 3.67**	20.76 ± 3.93**	0.608
IBDQ	14239.19	178 ± 44.24****	184.4 ± 34.51****	0.0010

All values are mean ± SD unless high ESR and high CRP. High ESR > 20 mm/h; High CRP > 0.8 mg/dl. *p < 0.05, **p < 0.01, ****p < 0.0001 (Paired *t* test with GraphPad Prism 7.00 and Chi-Square Test with IBM SPSS Statistics 23 versus F0).

During a 4-year follow-up, the mean remission duration was 26.5 ± 19.98 m (3 m∼48 m). Among 12 patients with remission after FMT, 4 patients (33.33%) relapsed within one year, and 6 patients (50%) relapsed within two years. Notably, four participants remained in remission for four years without receiving medication even mesalamine.

### Adverse Events

A portion of patients experienced mild adverse events shortly after FMT. Low fever was the most common side effect (27.4%, 17/62), followed by abdominal pain (9.7%, 6/62) and transient abdominal distension (9.7%, 6/62) (Table 4). Most adverse events were transient and disappeared spontaneously within hours.

**TABLE 4:** Safety assessment of 31 patients with 62 FMTs.

**Adverse effects**	**Proportion**	**Duration**	**Treatment**	**Notes**
Fever	27.4% (17/62)	A few hours ∼1 day	Spontaneous relief or physical cooling	The body temperature was approximately 37.5°C, the highest temperature was 39°C, which decreased to normal level the second day without chills.
Abdominal pain	9.7% (6/62)	A few hours	One patient had persistent abdominal cramps after administration via gastroscopy and achieved remission after the administration of intramuscular anisodamine. The others had mild symptoms.	
Abdominal distension	9.7% (6/62)	1∼3 days	Spontaneous relief	Mostly mild
Nausea	1.6% (1/62)	A few hours	Spontaneous relief	
Furuncle in the leg	1.6% (1/62)	1 week	No diffusion, scab without treatment	

### Bacterial Analysis

**FIGURE 3:**
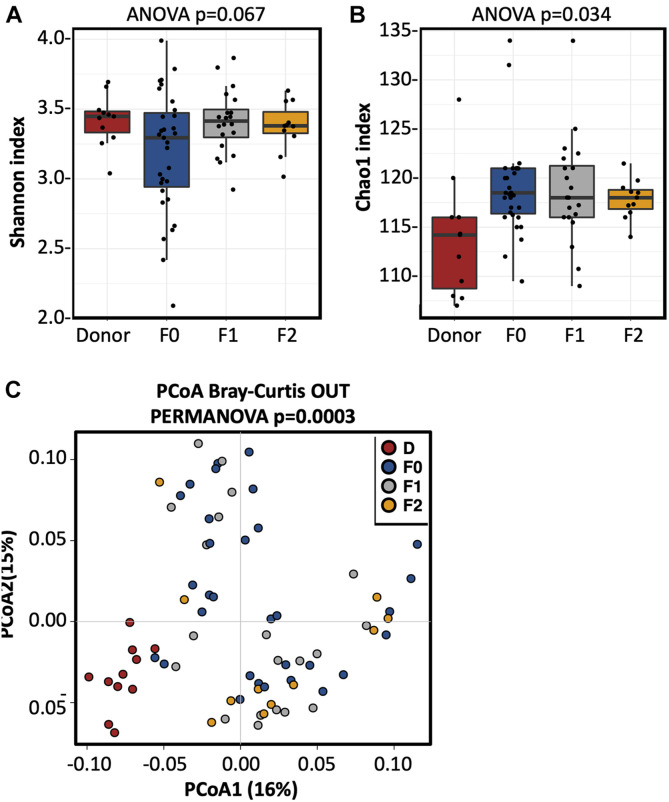
The fecal microbiota alpha diversity increased in the UC patients after FMT. **(A)** Shannon index and **(B)** Chao1 index of the fecal microbiota in UC patients prior and after FMT treatments. ANOVA is performed to evaluate alpha diversity among the different groups. **(C)** PCoA analysis of the fecal microbiota in donor and UC patients prior and after FMT. Statistical significance of distances among the four groups was assessed using PERMANOVA. D, donor; F0, before FMT; F1, 2∼3 months after the first FMT, or before the second FMT; F2, 2∼3 months after the second FMT.

Seventy-three fecal samples were collected, 11 of which were from the single donor (Donor 3), 31 from the UC patients at F0, 20 from the corresponding recipients at F1, and 11 were from the corresponding recipients at F2. The bacterial alpha diversity (measured by Simpson’s index and Shannon index) of UC patients was significantly lower compared to the donor at baseline (F0), but increased to the donor level after FMT treatment (Figures 3A,B). The results of principal component analysis (PCoA) analysis suggested that the microbial community composition and structure of UC patients were shifted toward a donor-like state after the first FMT, and this transformation was enhanced after the second FMT (Figure 3C).

**FIGURE 4:**
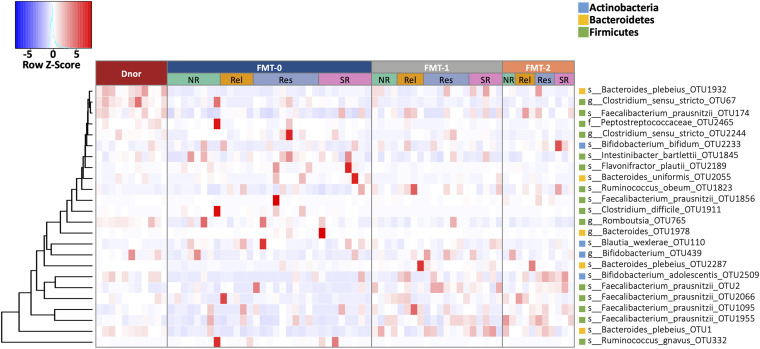
The fecal microbiota composition of UC patients shifted by FMT. OTUs that were statistically different in abundance between F0 and F1, and between F0 and F2 (Wilcoxon rank-sum test *p* < 0.01). Heatmap is color-coded based on row z-scores. D, donor; F0, before FMT; F1, 2∼3 months after the first FMT, or before the second FMT; F2, 2∼3 months after the second FMT.

Among the top 100 most abundant OTUs in the fecal samples of UC patients at the baseline, the relative abundances 55 OTUs were significantly different from that of the donor (Mann Whitney *U* test *p* < 0.05; Supplementary Table 4). Among the UC-enriched taxa, we observed some potential pathogens that reported to induce/exacerbate inflammation in IBD, such as *Bacteroides fragilis* (Rabizadeh et al., 2007), *Clostridium difficile* (Negron et al., 2016), and *Ruminococcus gnavus* (Hall et al., 2017). In contrast, multiple short-chain fatty acid (SCFA) producing Firmicutes taxa were significantly depleted in UC such as *Eubacterium hallii*, *Faecalibacterium prausnitzii*, and *Roseburia* spp. (Louis and Flint, 2017). The composition of the gut bacterial community significantly changed in the patients after FMT treatment (Figure 4; Supplementary Table 5). Among the FMT-increased OTUs, six belong to *Faecalibacterium prausnitzii*, five to genus *Bifidobacterium*, and four to *Bacteroides plebeius*. Moreover, some potential pathogenic microorganisms such as *Clostridium difficile* and *Ruminococcus gnavus* were substantially decreased after FMT (Supplementary Table 5).

To further analyze the associations of the fecal microbiota with different therapeutic outcomes, we performed Spearman correlations between the relative abundance of fecal bacteria and some clinical variables including Mayo score, IBDQ, stool frequency, erythrocyte sedimentation rate (ESR), C-reactive protein (CRP), white blood count (WBC), and neutrophil (Supplementary Table 6). The relative abundance of *g__Bifidobacterium_OTU1617* was inversely correlated with the Mayo score, stool frequency, ESR, and neutrophil, indicating its pleiotropic effect in improving the UC symptoms. In contrast, *s__Nocardia_coeliaca_OTU2421* was found to be positively correlated with the Mayo score, stool frequency, and neutrophil, implicating a its pathogenic potential for UC.

**FIGURE 5:**
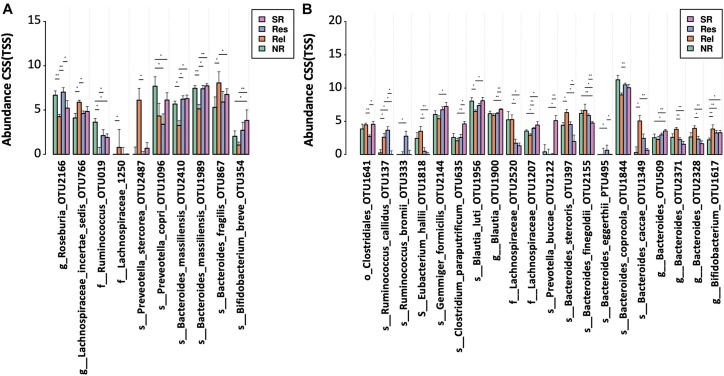
Bacterial taxa associated with different FMT outcomes. OTUs were compared among the patient with different therapeutic outcomes **(A)** before and **(B)** after FMT. Significance was determined by Kruskal-Wallis test (*p* < 0.05) and confirmed by pairwise Mann-Whitney *U* test (*p < 0.05 and **p < 0.01) NR, no response; Rel, relapse; Res, responders; SR, sustained remission.

Based on the therapeutic outcomes, we classified the UC patients into the SR (SR, sustained remission patients without relapse in 4 years), Res (Res, responders to FMT without relapse in 4 years), Rel (remission or response patients relapsed within 4 years), and NR (NR, patients with no response) groups. In prior to FMT (F0), NR and Rel patients showed a lower microbial alpha diversity (measured in Shannon’s index) than Res and SR patients, albeit significance was not achieved (Supplementary Figures 2A,C). In addition, the Rel patients were characterized by higher abundances of *Bacteroides fragilis* and *Lachnospiraceae incertae sedis*, together with lower abundances of *Bacteroides massiliensis*, *Roseburia*, and *Ruminococcus*; *Prevotella copri* was more abundant in the NR patients; and the SR patients had a higher abundance of *Bifidobacterium breve* (Figure 5A).

After FMT, the alpha diversity reached similar levels across the groups with different therapeutic response (Supplementary Figures 2B,D). NR patients had a lower level of *Bifidobacterium* compared to other response groups (Figure 5B). A higher level of multiple *Bacteroides* spp. (including OTU232, OTU397, OTU1349 and OTU2371) was observed in the Rel patient group, while the SR patients had a lower level of *Bacteroides finegoldii*, and a higher level of some *Blautia* (OTU1900). In addition, a reduced level of taxon (OTU1641) belonging to Clostridiales order was observed in the Res patients.

## Discussion

Differences in FMT procedures might cause different therapeutic effects in various studies. Most previous studies including RCTs performed FMT with high intensities (pooled donors and frequent treatments over a short duration). FMT with a single donor and long interval between administrations could be considered as a low intensity regimen. Although UC patients benefit from FMT, concerns about the efficacy, safety, and durability of low-intensity FMT for UC treatment remains to be addressed. In this study, we performed FMT using stool suspensions from a single donor with time interval of 2 months for all UC patients, and achieved a remission rate of 22.58% (7/31) after the first FMT and 60% (12/20) after the second FMT. All the clinical indicators including Mayo scores, Endoscopic mayo scores, hematochezia scores, stool frequency, IBDQ were improved after the two FMT treatments. Thus, our results suggested that a low-intensity single donor FMT can lead to positive therapeutic results.

The long-term outcomes and health consequences of FMT remain to be established, with most studies reporting mixed results and without showing a sustained benefit of FMT (Brandt et al., 2012; Kump et al., 2013; Vermeire et al., 2016). Analysis with 109 UC patients received FMT from two clinical trials (NCT01790061, NCT02560727), 21.1% (23/109) and 25.7% (28/109) of patients maintained clinical response at 6 months after single FMT, and step multiple FMTs, respectively (Ding et al., 2019). In another study (Sood et al., 2019a), that the primary outcome of maintenance of clinical remission at 48 weeks was achieved in 27/31 [87.1%] patients who received FMT via colonoscopy every 8 weeks. In the present study, the longest remission time was 6 years which continued till this article was completed, and in 8 of the 12 patients (66.7%) in clinical remission, the results remained in remission at 1-year follow up, 50% (6/12) at 2-year follow up and 41.7% (5/12) at 4-year follow up, without continued fecal infusion. Nevertheless, UC patients also have recurrence issues after FMT treatment or other therapies, and some studies reported maintenance FMT administration could sustain clinical efficacy either by capsule or colonoscopy (Sood et al., 2019a; Steube et al., 2019). Long-term maintenance treatment is necessary for some patients.

In one retrospective study regarding the long-term safety of FMT to treat UC, new-onset urticaria, arthritis, depression, and so on, were found in the long-term follow-up, while abdominal discomfort, flatulence, low-grade fever were the most common short-term adverse events (Sood et al., 2019b). Besides, one study reported a serious adverse event of myasthenia gravis in 1 month after FMT treatment (Ding et al., 2019). In our study, all the patients treated with FMT were observed for 4 years, with a maximum of 6.5 years. During the follow-up period, short-term adverse effects were consistent with previous studies by and large, but no long-term adverse reactions caused by FMT were found. Nevertheless, a remarkable adverse event was that one 14-year-old girl underwent furuncle in her legs after the first FMT treatment, though the furuncles scabbed in 1 week without any treatment. The risk of infectious disease transmission for FMT needs to be investigated with more cases collected. On June 13, 2019, the US FDA issued a safety alert regarding two immunocompromised adults who acquired Extended Spectrum Beta-Lactamase (ESBL)-producing *E. coli* infections following FMT, resulting in one death. Hence, the donor screening protocols for FMT should be further improved by excluding use of stool that tests positive for multi-drug resistant organisms.

Diverse administration routes of FMT have been observed to have similar efficacy in the studies of FMT for the treatment of CDI (Postigo and Kim, 2012; Furuya-Kanamori et al., 2017). We used a combination of colonoscopy and endoscopy as the route of FMT for 11 patients with a clinical response rate 63.6%, and the next 20 patients underwent only a colonoscopy approach, who also experienced good efficacy with a clinical response rate 70%. In the studies of FMT treatment for UC, colonoscopy approach, enema and nasogastric tube are the most common methods of administration. In the five RCT studies, four obtained positive results by conducting under lower GI tract administration (Moayyedi et al., 2015; Paramsothy et al., 2017; Costello et al., 2019), and one obtained negative results by using upper gastrointestinal tract route (Rossen et al., 2015). It is speculated that the upper gastrointestinal administration might be one of the reasons leading to the treatment failure, and FMT via colonoscopy route may be a better choice for UC.

Previous studies mainly focused on mild to moderate UC, including the above-mentioned RCT studies. A few cohort studies have reported the efficacy of FMT on moderate to severe UC (Angelberger et al., 2013; Kump et al., 2013; Ding et al., 2019), and showed only marginally clinical responses. In our study, patients with moderate UC had the highest responses to FMT, followed by, in turn, severe and mild UC. It is worth noting that the FMT treatment was more effective in patients with extensive UC, followed by distal UC. And patients with proctitis had poor responses to FMT, while some patients with extensive and distal UC responded to FMT but were more likely to leave behind lesions in rectum and/or left-sided colon. These findings suggest that lesions confined to distal colon, especially proctitis, had less responsive to FMT and may require more long-term treatment (persistent therapy). It was recently suggested that CD patients with a low microbial load presented a better response to FMT (Sarrabayrouse et al., 2020). Moreover, oral decontamination with antibiotics such as colistin or aminoglycosides has been proposed to enhance the efficacy of FMT (Huttner et al., 2019; Saidani et al., 2019). Thus, an initial low microbial load or reducing the microbial load in UC patients may promote the colonization of donor microbiota thereby enhancing the efficacy of FMT. In fact, the bacterial load various at different location of the GI tract, with a higher bacteria load in the distal colon (∼10^10^CFU/ml) than the ileum (∼10^6^CFU/ml), which give rise to different degrees of colonization resistance. Therefore, an intensive FMT regime may be required to efficiently modulate the microbiome for UC patients with the distal colitis.

The gut microbiota profile analysis demonstrated similar results as reported in previous studies of FMT (Costello et al., 2019; Paramsothy et al., 2019). For instance, a lower gut microbiota diversity was observed in the UC patients compared with the healthy donor, which was increased after FMT treatments. The composition of the gut bacterial community was shifted by FMT to a donor-like state and was enhanced after the second administration. These results suggest that repeated FMT treatments enhance the colonization of donor-derived microbiota in UC patients leading to improvement in clinical symptoms. In particular, *Faecalibacterium prausnitzii*, *Bacteroides plebeius*, and *Bifidobacterium* spp. were significantly increased in UC patients receiving FMT, agreeing with some previous studies (Lopez-Siles et al., 2017; Nishino et al., 2018; Lloyd-Price et al., 2019). Interestingly, an increased level of some *Bifidobacterium* spp. was closely correlated with a reduction in Mayo score, stool frequency, ESR, and neutrophil, indicating a pleiotropic of *Bifidobacterium* for UC symptoms improvement (Jakubczyk et al., 2020).

As one of the main butyrate producers in the gut, *Faecalibacterium prausnitzii* is able to induce secretion of anti-inflammatory cytokines, thereby producing energy to the colonocytes and enhancing the intestinal barrier (Sokol et al., 2008; Louis and Flint, 2009; Ferreira-Halder et al., 2017; Lopez-Siles et al., 2017). Hence, the relative abundances of *Faecalibacterium prausnitzii* increased in UC patients after FMT treatments, suggesting a potential contribution in suppressing inflammation. In addition, we found that a high abundance of *Blautia* in UC patients at baseline was associated with sustained remission, and an enrichment of *Roseburia* was particularly associated with therapeutic responses. These results were consistent with previous studies (Paramsothy et al., 2019; Lloyd-Price et al., 2019).

Data about the correlation between *Bacteroidetes* spp. abundance and UC activity was controversial. In fact, different *Bacteroides* spp. may have different influences on the disease development in UC patients. For example, *Bacteroides plebeius* showed a significant therapeutic effect in UC patients from our study, whereas their nearest recognized species *Bacteroides vulgatus* (Kitahara et al., 2005) was considered as a pathobiont in human gut (Kootte et al., 2017)with a potential of driving injury in the small intestine (Ramanan et al., 2014). From our data, a combination of reduced *Bacteroides massiliensis* and enriched *Bacteroides fragilis* seemed to augur a relapse in UC after FMT. *Bacteroides fragilis* was shown to play a protective role from intestinal inflammation via Toll-like receptor 2 signaling, inducing production of polysaccharide A (PSA) and anti-inflammatory cytokine IL-10 (Mazmanian et al., 2008; Honda and Littman, 2016; Lee et al., 2018). Nevertheless, strains of enterotoxigenic *Bacteroides fragilis* have functions of tissue invasion and induce severe intestinal inflammation in humans and animals (Zamani et al., 2017). Moreover, a lower level of *Bacteroides finegoldii* after FMT characterized the patients with sustained remission. In contrast, Paramsothy et al. (2019) found that an increased abundance of *Bacteroides finegoldii* in donor stool may be associated with observed remission in UC patients receiving FMT. Thus, close species may have significantly distinct characteristics and the same species may contain various strains with different functions. Moreover, our data indicated that *Blautia* spp. and *Ruminococcus bromii* were predictors of achieving the sustained remission, which is in line with the findings in Paramsothy et al. (2017, 2019).

*Preveotella copri* was found to be more abundant in the non-responders at baseline. In a previous study (Paramsothy et al., 2019), *P. copri* was also found in higher abundance in UC patients who experienced therapeutic failure after FMT. The relative abundance of *P. copri* presents more stable in the population with IBD than non-IBD populations (Lloyd-Price et al., 2019). It was postulated that *Prevotella* has an antagonistic relationship with *Bacteroides* (Ley, 2016). Thus, the response of FMT might be partially impeded by *P. copri* resisting the colonization of the donor bacteria such as *Bacteroides*. Moreover, *P. copri* was suggested to play an immune-modulatory role in human rheumatoid arthritis (RA). About 32% of patients with RA were found to have serum immunoglobulin A (IgA) antibodies specific for *P. copri* (Larsen, 2017), which was almost absent in healthy subjects. Secretory IgA is the dominant immunoglobulin at the mucosal surface, and it plays a critical role in interacting with the microbiota and maintaining intestinal homeostasis. It was suggested that IgA coating identifies colitogenic bacteria in IBD patients and is associated with treatment outcomes (Palm et al., 2014; Shapiro et al., 2021). Therefore, *P. copri*-specific IgA may be related to poor response to FMT for UC patients, which deserves further investigation.

This is a series study with only 31 cases at baseline, which were further reduced at the points of F1 (*n* = 20). Therefore, this small cohort size and samples limited the strength of our conclusions. In addition, there was a loss of endoscopic examination in the long-term follow-up.

Collectively, our study demonstrated that low-intensity single donor FMT treatment is effective and safe for mild to severe UC, and a repeated FMT provides a beneficial efficacy for disease improvement, with a considerable long-term efficacy and safety. The abundances of *Faecalibacterium prausnitzii*, *Bacteroides plebeius* and *Bifidobacterium* spp. increased significantly after FMT treatments in UC patients. The gut microbial composition of UC with different FMT therapeutic outcomes was distinguishable at baseline. In particular, a higher level of *Bacteroides fragilis* together with lower levels of *Bacteroides massiliensis* and *Roseburia* genus might be indicators of relapse given this particular donor. Moreover, an enrichment of *Blautia* spp. and *Ruminococcus bromii* together with a reduced *Bacteroides finegoldii* were associated with long-term remission. Further investigations to identify specific species/strains associated with clinical remission may shed a light for precise treatment, and a donor-recipient matching approach based on the gut microbiota compositions may increase the remission rate in long term.

## Data Availability Statement

The original contributions presented in the study are included in the article/Supplementary Material, further inquiries can be directed to the corresponding author/s.

## Ethics Statement

The studies involving human participants were reviewed and approved by the Chinese PLA General Hospital Ethics Committee. Written informed consent to participate in this study was provided by the participants’ legal guardian/next of kin. Written informed consent was obtained from the individual(s), and minor(s)’ legal guardian/next of kin, for the publication of any potentially identifiable images or data included in this article.

## Author Contributions

YY responsible for the study concept and design. RR analysis and interpretation of the clinical data and drafted the manuscript. XG analysis and interpretation of the microbiome data and critical revision of the manuscript. YS, JL, LP, GS, ZW, BY, and JZ performance of the FMT and revision of the manuscript. All authors had access to the study data and have reviewed and approved the final manuscript.

## Conflict of Interest

The authors declare that the research was conducted in the absence of any commercial or financial relationships that could be construed as a potential conflict of interest.

## Publisher’s Note

All claims expressed in this article are solely those of the authors and do not necessarily represent those of their affiliated organizations, or those of the publisher, the editors and the reviewers. Any product that may be evaluated in this article, or claim that may be made by its manufacturer, is not guaranteed or endorsed by the publisher.

## Supplementary Material

Supplementary Figure 1 Improvement of intestinal mucosa lesions and histopathological images of PUC2 after two FMT treatments. F0, before FMT; F1, 2 months after the first FMT; F2, 5 months after the first FMT. PUC2, 40y, male, disease course: 15 years, extensive UC (E3), glucocorticoids (60 mg) for 2 weeks combined with mesalamine (4 g) more than 4 weeks did not respond well.

Supplementary Figure 2 Comparisons of alpha diversity among UC patients with different FMT outcomes. Shannon index and Chao1 index are compared among UC patients **(A,B)** at baseline as well as **(C,D)** post-FMT with one-way ANOVA. NR, no response; Rel, relapse; Res, responders; SR, sustained remission.
