# Supplementary figures and images for: Long-Term Efficacy of Low-Intensity Single Donor Fecal Microbiota Transplantation in Ulcerative Colitis and Outcome-Specific Gut Bacteria
Source: Front Microbiol. 2021 Nov 17;12:742255. doi: 10.3389/fmicb.2021.742255 (PMC8635752; doi:10.3389/fmicb.2021.742255)

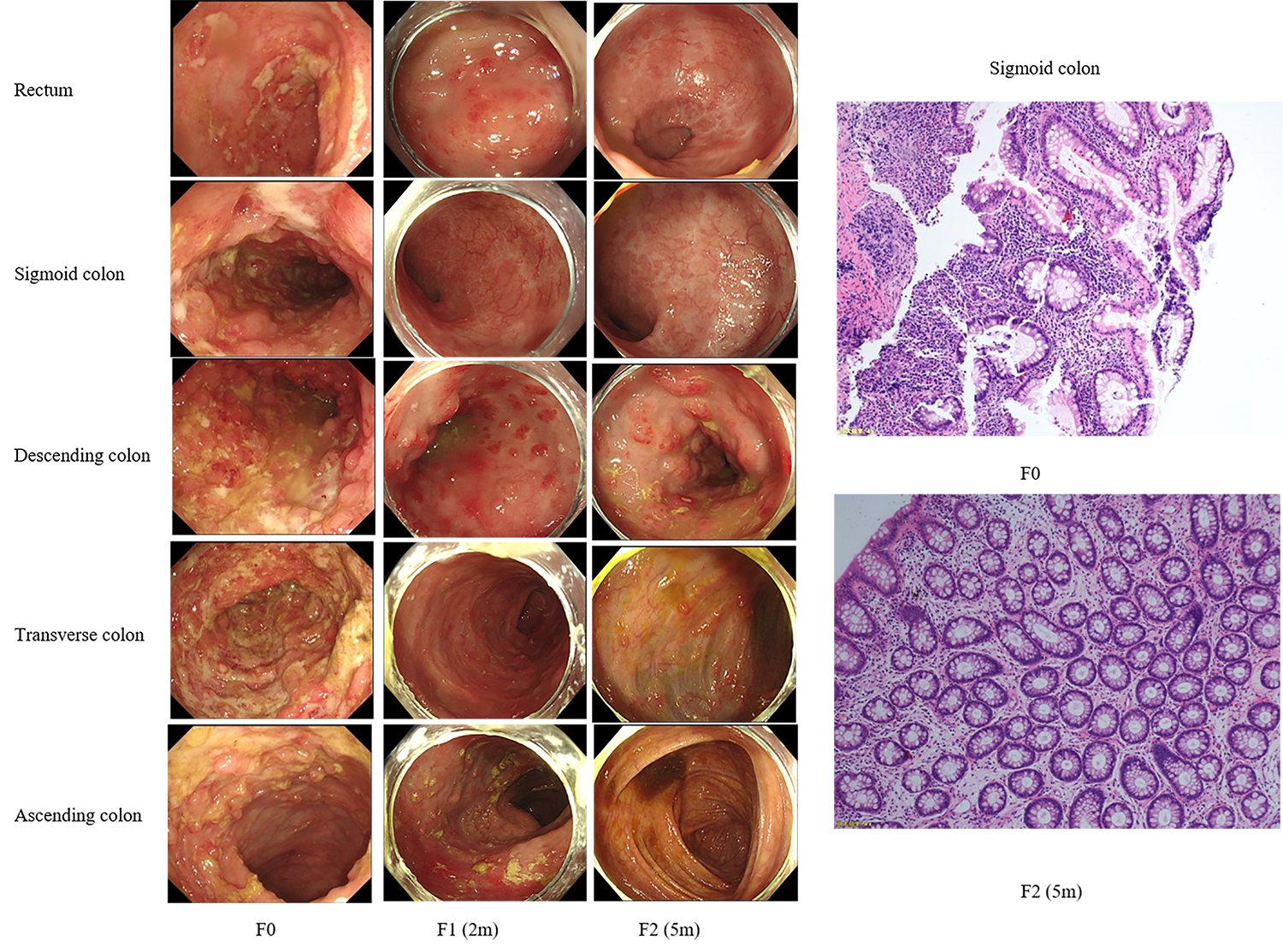

Supplement: Supplementary Figure 1 — Improvement of intestinal mucosa lesions and histopathological images of PUC2 after two FMT treatments. F0, before FMT; F1, 2 months after the first FMT; F2, 5 months after the first FMT. PUC2, 40y, male, disease course: 15 years, extensive UC (E3), glucocorticoids (60 mg) for 2 weeks combined with mesalamine (4 g) more than 4 weeks did not respond well. [file Image_1.TIF]

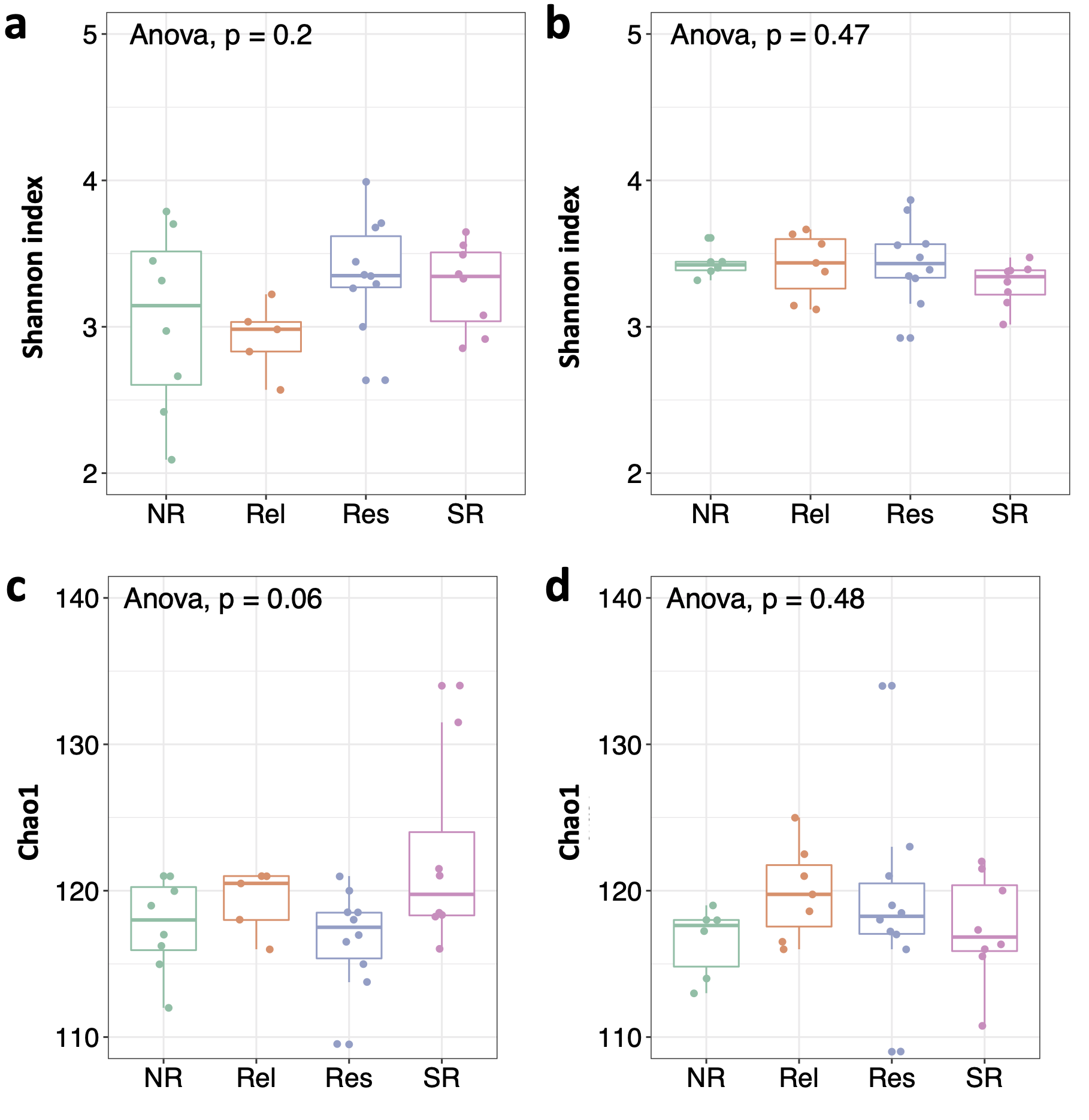

Supplement: Supplementary Figure 2 — Comparisons of alpha diversity among UC patients with different FMT outcomes. Shannon index and Chao1 index are compared among UC patients (A,B) at baseline as well as (C,D) post-FMT with one-way ANOVA. NR, no response; Rel, relapse; Res, responders; SR, sustained remission. [file Image_2.TIF]
